# Identifying Exifone as a Dual-Target Agent Targeting Both SARS-CoV-2 3CL Protease and the ACE2/S-RBD Interaction Among Clinical Polyphenolic Compounds

**DOI:** 10.3390/ijms26052243

**Published:** 2025-03-02

**Authors:** Jiani Lu, Yan Tang, Hongtao Li, Xixiang Chen, Pengcheng Qin, Jianrong Xu, Weihua Li, Lili Chen

**Affiliations:** 1 Shanghai Frontiers Science Center of TCM Chemical Biology, Institute of Interdisciplinary Integrative Medicine Research, Longhua Hospital, Shanghai University of Traditional Chinese Medicine, Shanghai 201203, China; genie_lu@163.com (J.L.); 13473346278@163.com (H.L.); 13213970082@163.com (P.Q.); 2 Shanghai Frontiers Science Center of Optogenetic Techniques for Cell Metabolism, Shanghai Key Laboratory of New Drug Design, School of Pharmacy, East China University of Science and Technology, Shanghai 200237, China; tangyan9420469@163.com; 3 Shanghai Frontiers Science Center of TCM Chemical Biology, Institute of Interdisciplinary Integrative Medicine Research, Academy of Integrative Medicine, Shanghai University of Traditional Chinese Medicine, Shanghai 201203, China; 22021505@shutcm.edu.cn (X.C.); janker.xu@gmail.com (J.X.); 4 School of Pharmacy, Henan University, Kaifeng 475001, China

**Keywords:** polyphenols, SARS-CoV-2 3CL^pro^, S-RBD, exifone, benserazide hydrochloride

## Abstract

The ongoing emergence of severe acute respiratory syndrome coronavirus 2 (SARS-CoV-2) variants has led to resistance against multiple coronavirus disease 2019 (COVID-19) vaccines and therapeutic medications, making the development of effective therapeutics against SARS-CoV-2 a high priority. Studies have shown that bioactive polyphenols, particularly those with triphenol groups, can effectively inhibit the activity of SARS-CoV-2 3-chymotrypsin-like protease (3CL^pro^). However, the structural instability of polyphenols necessitates further research. To address this, we conducted a literature review to identify triphenol compounds that are either approved or currently undergoing clinical trials, assessing their potential to inhibit SARS-CoV-2 3CL^pro^. Exifone and benserazide hydrochloride were identified as the inhibitors of SARS-CoV-2 3CL^pro^ among these compounds, using a fluorescence resonance energy transfer (FRET)-based assay. Benserazide hydrochloride was confirmed as a covalent binder to SARS-CoV-2 3CL^pro^ through time-dependent inhibition and kinetic analysis, with its binding mode elucidated by molecular docking. Notably, exifone not only inhibited the protease activity but also blocked the interaction between the host cell receptor angiotensin-converting enzyme 2 (ACE2) and the SARS-CoV-2 spike protein receptor binding domain (S-RBD), as identified by surface plasmon resonance (SPR) and flow cytometry. Additionally, exifone demonstrated antiviral activity against various SARS-CoV-2-S pseudovirus variants. In conclusion, the discovery of exifone and benserazide hydrochloride underscores the potential of polyphenols in developing conserved 3CL^pro^ inhibitors for coronaviruses, offering new strategies for the rapid development of effective drugs against both current and future coronavirus pandemics.

## 1. Introduction

The coronavirus disease 2019 (COVID-19) pandemic caused by Severe Acute Respiratory Syndrome Coronavirus 2 (SARS-CoV-2) and its constantly appearing variants continues to affect the health and daily lives of people around the world [1]. Therefore, the development of new drugs against SARS-CoV-2 remains an important endeavor.

Coronaviruses are a large family of viruses that include several deadly human viruses: Severe Acute Respiratory Syndrome Coronavirus (SARS-CoV) (2003), Middle East Respiratory Syndrome Coronavirus (MERS-CoV) (2012), and SARS-CoV-2 (2019) [2]. As one of the important non-structural proteins in coronaviruses, 3-chymotrypsin-like protease (3CL^pro^, also known as the main protease, M^pro^) plays a key role in the life process of the virus and has high conservation of amino acid sequence, particularly in its catalytic dyad composed of cysteine (Cys-145) and histidine (His-41) [3,4]. It is a cysteine protease that cleaves two SARS-CoV-2 polyproteins (pp1a and pp1ab) at 11 different sites, resulting in the production of various nonstructural proteins that are key to viral replication [5,6]. The substrate of 3CL^pro^ presents a unique glutamine structure at the P1 site (Leu-Gln/Ser, Ala, Gly), whereas no known human protease recognizes this cleavage site [7]. Several drugs have been marketed to combat COVID-19, including Paxlovid, Ensitrelvir, Simintrelvir, and others [8,9,10]. Thus, the essential role of 3CL^pro^ in viral replication, its high conservation across coronaviruses, and its unique substrate specificity, combined with the potent antiviral efficacy of its inhibitors such as Paxlovid, make it a highly attractive target for developing broad-spectrum antiviral drugs against SARS-CoV-2 and other coronaviruses.

In addition to 3CL^pro^, the trimeric spike (S) protein consisting of S1 and S2 subunits is essential for viral entry into the host cells [11]. The receptor binding domain of the S1 subunit (S-RBD) can bind to the host cell receptor angiotensin-converting enzyme 2 (ACE2), causing a conformational change of the S2 subunit and thus mediating virus fusion to the cell membrane of the host cells [12,13,14]. Thus, S-RBD/ACE2 is another important target for the screening and discovery of active drugs against SARS-CoV-2. Inhibitors of protein–protein interaction between S-RBD/ACE2 have recently attracted increasing attention for the development of potential antiviral drugs to prevent viral attachment and cellular entry.

Previous studies have shown bioactive polyphenols could effectively inhibit SARS-CoV-2 3CL^pro^ activity. Compounds such as epigallocatechin gallate, curcumin, resveratrol, quercetin, and ellagic acid exhibited significant inhibitory activity in both in silico and in vitro studies [15]. The pyrogallol group in myricetin forms a covalent bond with the catalytic cysteine of SARS-CoV-2 3CL^pro^, which was used as an effective warhead to design the compounds inhibiting the activity of SARS-CoV-2 3CL^pro^ [16]. Su et al. reported that the three phenolic hydroxyl groups of baicalein appeared to form hydrogen bonds, offering interaction advantages with 3CL^pro^ [17]. Looking back at our previous results, the polyphenolic group also plays an important role in inhibiting SARS-CoV-2 3CL^pro^ activity [18]. At the same time, combining the results of several literatures with our experimental results, it is obvious that the triphenol compound has better inhibitory activity against SARS-CoV-2 3CL^pro^ than those compounds with two phenolic hydroxyl groups (Table 1). In addition, several studies have investigated the role of polyphenols in inhibiting the interaction between S-glycoprotein of receptor-binding domain (RBD) and ACE2. A previous publication has reported that pomegranate extracts rich in polyphenols, the major polyphenols and their major metabolite, including punicalin, punicalagin, and urolithin, could block the interaction between s-glycoprotein and ACE2 [19]. Since the phenolic hydroxyl group is structurally unstable and not easy to become a drug [20], we conducted a literature review to identify triphenol compounds that are either approved or in clinical trials. Then we assessed their inhibitory activities against SARS-CoV-2 3CL^pro^ using a well-established screening method (Table 2).

Here, among the five purchased compounds, exifone and benserazide hydrochloride exhibited potent inhibitory activities against SARS-CoV-2 3CL^pro^ by a fluorescence resonance energy transfer (FRET)-based assay. To clarify the mechanisms of action for the two compounds, we conducted time-dependent inhibition (TDI) and kinetic analyses. These studies showed that benserazide hydrochloride is covalently bound to SARS-CoV-2 3CL^pro^. In addition, we verified the binding mode of benserazide hydrochloride by molecular docking, providing detailed mechanistic insights into the covalent mode of action. Notably, exifone not only inhibited the protease activity but also blocked the entry of SARS-CoV-2-S pseudovirus into hACE2-HEK293T cells. In addition, exifone showed inhibitory activity against various SARS-CoV-2 pseudovirus variants. Exifone could block the interaction between the purified his-S-RBD and ACE2-expressing Vero-E6 cells detected by flow cytometry. Furthermore, to explore the underlying anti-SARS-CoV-2 mechanism of action, surface plasmon resonance (SPR) was performed to detect the strong binding affinity between exifone and ACE2 or S-RBD. Therefore, exifone is a newly discovered dual-target inhibitor with the potential to be developed as a potential antiviral drug against SARS-CoV-2 infection. Consistent with previous studies in other viral diseases [21,22,23,24], this dual-target therapeutic regimen for COVID-19 may prove to be more beneficial, greatly reducing combination agents and the multi-dose burden on the host system. Taken together, the discovery of exifone and benserazide hydrochloride highlights the potential of polyphenols for the discovery of conserved coronavirus 3CL^pro^ inhibitors, providing new ideas for the rapid development of effective drugs against the current and future coronavirus pandemics.

**Table 1:** Comparison of the activity of polyphenols against SARS-CoV-2 3CL^pro^ between the data from the literature and the results from our laboratory.

Data from the Literature					Data from Our Laboratory				
Number	Structure	Generic Name	IC_50_ (μM)	Ref.	Number	Structure	Generic Name	IC_50_ (μM)	Ref.
1	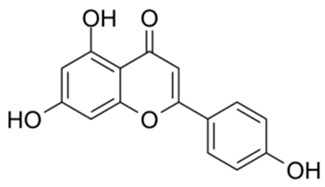	Apigenin	84.94	[25]	1	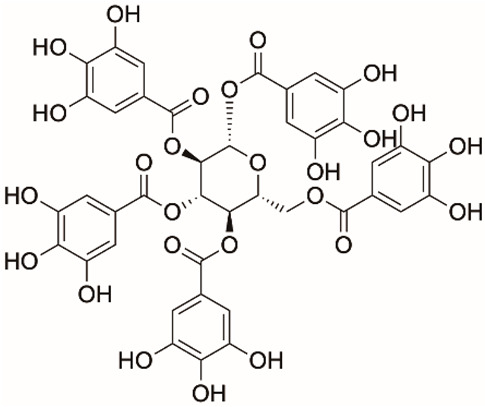	Pentagalloylglucose	3.88	[26]
2	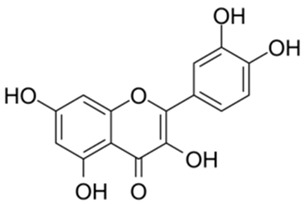	Quercetin	12.65	[25]	2	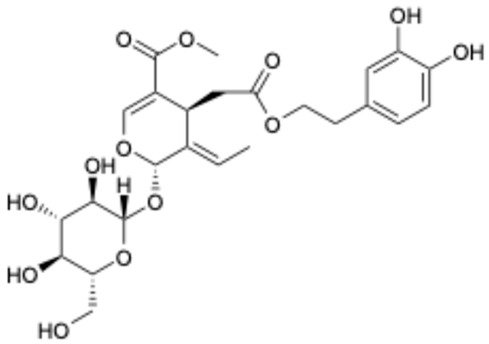	Oleuropein	3.50	
3	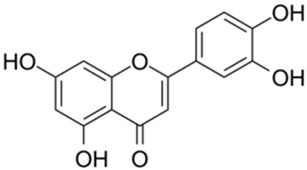	luteolin	74.86	[25]	3	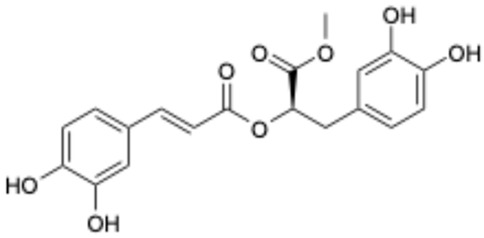	Methyl rosmarinate	2.50	
4	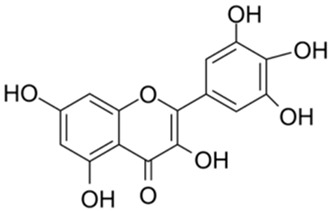	Myricetin	0.63	[27]	4	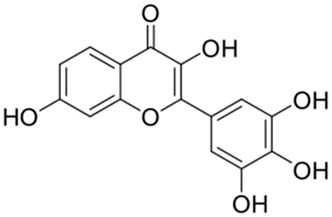	Robinetin	0.96	
5	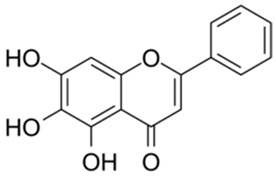	Baicalein	0.63	[27]	5	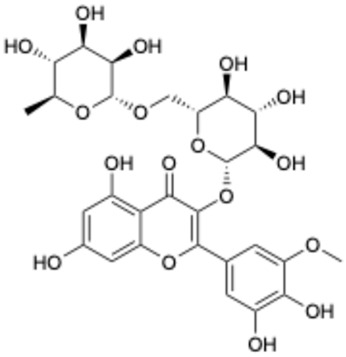	Laricitrin 3-rutinoside	3.82	Unpublished data
6	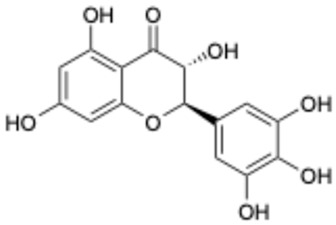	Dihydromyricetin	1.14	[27]	6	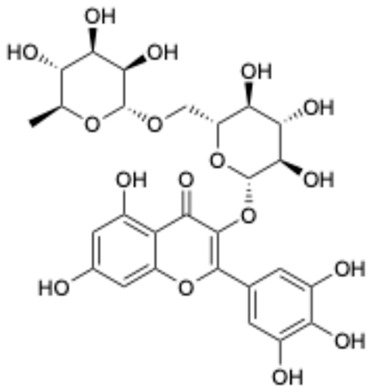	Myricetin-3-*O*-rutinoside	0.85	
7	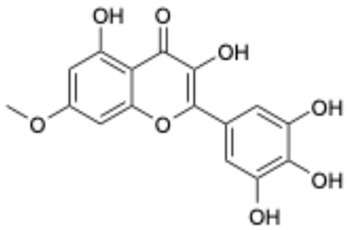	Compound 3	0.30	[27]	7	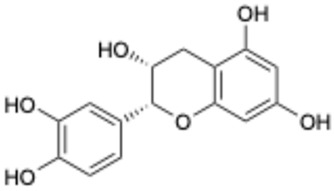	Epicatechin	3.30	
8	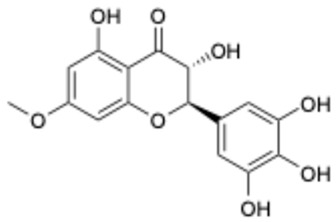	Compound 7	0.26	[27]	8	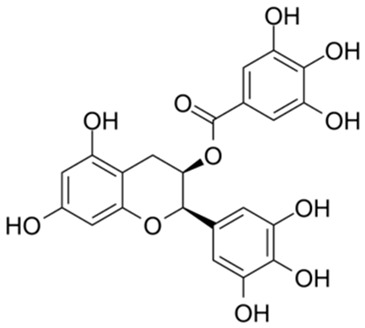	EGCG	0.30	
9	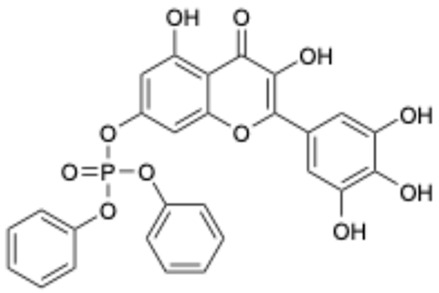	Compound 9	3.13	[27]	9	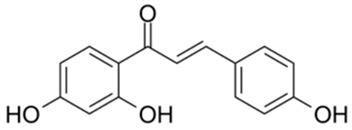	Isoliquiritigenin	73.92	
10	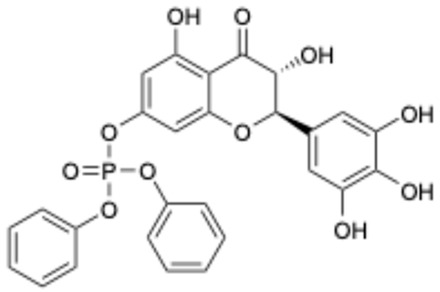	Compound 10	1.84	[27]					

**Table 2:** Polyphenolic compounds that have been approved or tested in clinical trials.

Number	Structure	Generic Name	CAS Number	Indications	Phase	Ref.
1	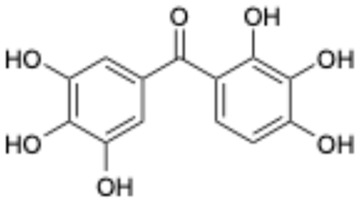	Exifone	52479-85-3	Cognitive problems in Parkinson’s disease	Withdrawal ^a^	[28]
2	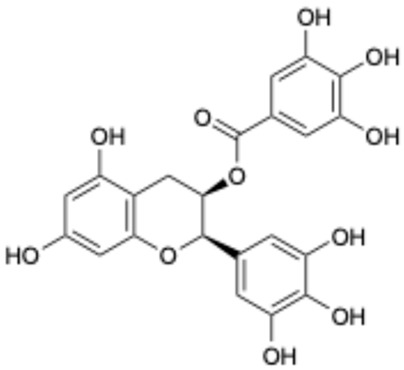	Epigallocatechin Gallate	989-51-5	Multiple sclerosis, Multiple system atrophy, Duchenne muscular dystrophy	Phase II/III	[29,30,31,32]
3	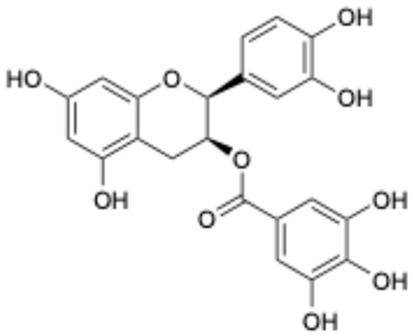	Epicatechin-3-O-gallate	1257-08-5	Prostate cancer prevention, etc.	Phase II	[33,34]
4	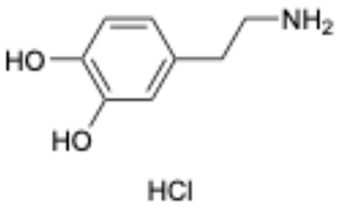	Dopamine hydrochloride	62-31-7	Hypotension, shock, and heart failure	Approved	[35]
5	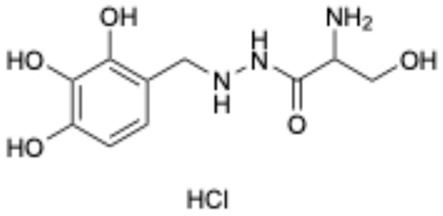	Benserazide hydrochloride	4919-77-8	Parkinson’s disease	Approved	[36,37]
6	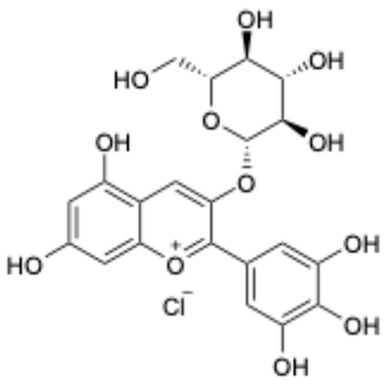	Delphinidin-3-glucoside	6906-38-3	Hyperlipidemia, etc.	Phase I	[38,39]
7	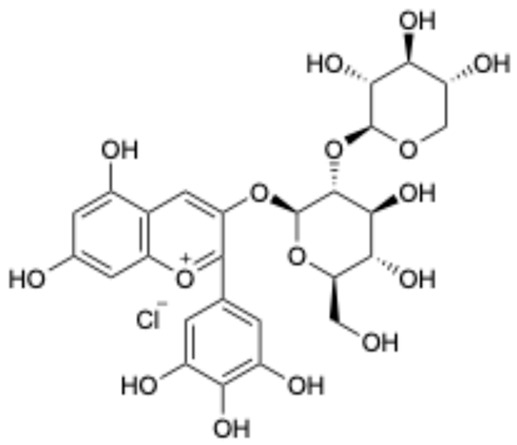	Delphinidin-3-sambubioside	53158-73-9	Hypertension	Phase I	[40]
8	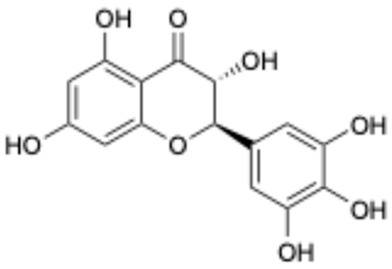	Dihydromyricetin	27200-12-0	Nonalcoholic fatty liver disease	Phase I	[41]
9	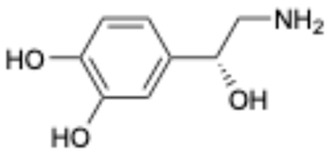	Norepinephrine	51-41-2	Septic shock, etc.	Approved	[42,43]

^a^ The withdrawal of exifone was primarily due to its hepatotoxicity [44].

## 2. Results and Analysis

### 2.1. Inhibition of Five Clinical Drugs Against SARS-CoV-2 3CL^pro^

**Figure 1:**
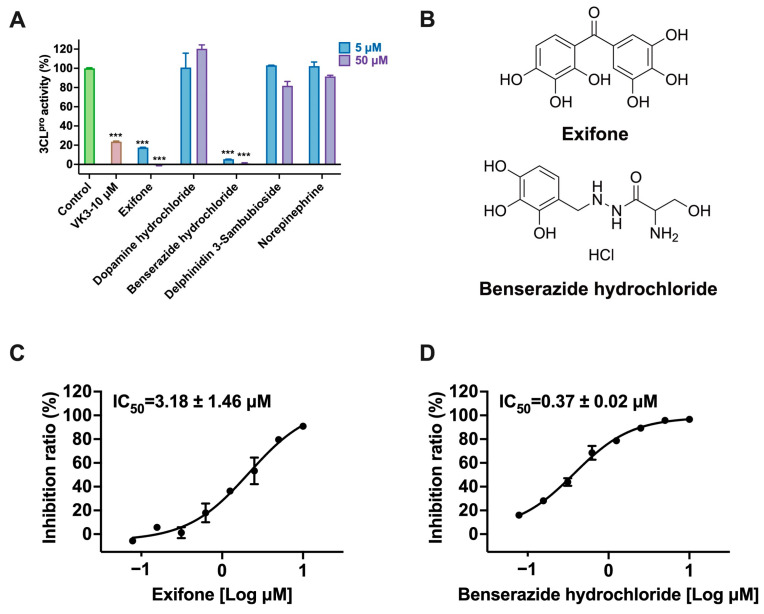
Inhibitory effects of the purchased polyphenolic compounds against SARS-CoV-2 3CL^pro^. (**A**) A preliminary screening was performed to evaluate the inhibitory activity of five clinical drugs at concentrations of 5 and 50 μM against SARS-CoV-2 3CL^pro^. (**B**) The chemical structures of two polyphenols, exifone and benserazide hydrochloride. (**C**,**D**) The inhibition curves depicted here represent the inhibitory effects of exifone and benserazide hydrochloride on SARS-CoV-2 3CL^pro^. Each data point represents the mean ± SEM of three independent experiments. Statistical differences were analyzed and determined based on *p*-values (*** *p* < 0.001).

Based on the results in Table 1, we speculated that the triphenol compounds exhibit better inhibitory activities against SARS-CoV-2 3CL^pro^ than the compounds with two phenolic hydroxyl groups. To quickly find the triphenol compounds with better druggability, we found nine triphenol compounds that have been approved or are currently under clinical trials. We procured five of them to assess their inhibitory activities against SARS-CoV-2 3CL^pro^ and chosen VK3 as a positive control for inhibiting 3CL^pro^ activity in the experiments. As shown in Figure 1A, among of five compounds, exifone and benserazide hydrochloride displayed significant inhibitory effects on SARS-CoV-2 3CL^pro^ activity (>50% inhibition at 5 μM). The chemical structures of two active compounds are presented in Figure 1B. To further investigate their inhibitory potency, we determined their half-maximal inhibitory concentrations (IC_50_), which were 3.18 μM for exifone and 0.37 μM for benserazide hydrochloride, respectively (Figure 1C,D). Considering their excellent enzyme inhibitory capabilities and established safety for human use, exifone and benserazide hydrochloride were chosen for further investigation.

### 2.2. Mechanisms of Exifone and Benserazide Hydrochloride Against SARS-CoV-2 3CL^pro^

**Figure 2:**
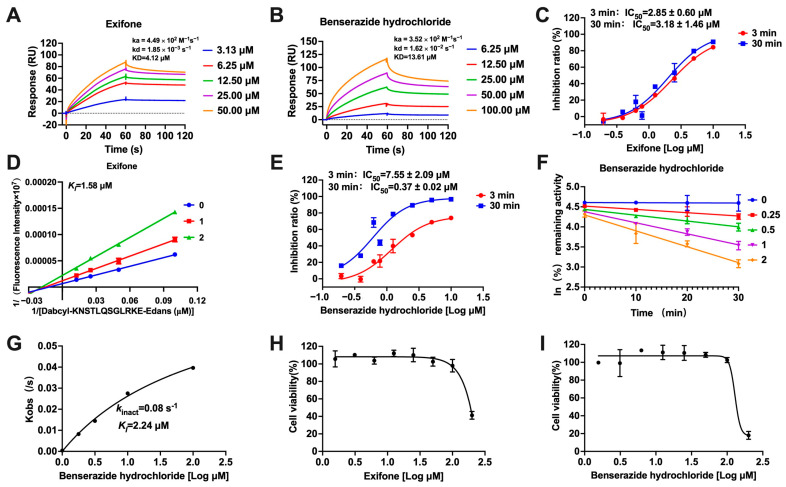
Elucidating the inhibition mechanisms of exifone and benserazide hydrochloride on SARS-CoV-2 3CL^pro^. (**A**,**B**) The binding affinities of exifone and benserazide hydrochloride to SARS-CoV-2 3CL^pro^ were evaluated using SPR analysis. (**C**) TDI assays were conducted to determine the time-dependent inhibitory effect of exifone on SARS-CoV-2 3CL^pro^. (**D**) The kinetic analysis of SARS-CoV-2 3CL^pro^ inhibition by exifone was depicted through Lineweaver–Burk plots. (**E**) The time-dependent inhibitory effect of benserazide hydrochloride on SARS-CoV-2 3CL^pro^ was depicted. (**F**,**G**) The concentrations of benserazide hydrochloride and *kobs* of SARS-CoV-2 3CL^pro^ inhibition were illustrated in hyperbolic plots. (**H**,**I**) The cytotoxic effects of exifone and benserazide hydrochloride on Vero-E6 cells were determined.

To further substantiate the interaction of exifone and benserazide hydrochloride with SARS-CoV-2 3CL^pro^, we employed SPR experiments to confirm their binding capability. As shown in Figure 2A, the equilibrium constant (KD) value between exifone and SARS-CoV-2 3CL^pro^ was 4.12 μM, with a binding rate constant (ka) and dissociation rate constant (kd) of 4.49 × 10^2^ M^−1^s^−1^ and 1.85 × 10^−3^ s^−1^, respectively. The study demonstrated that the calculated KD value of benserazide hydrochloride was 13.61 μM, with the ka of 3.52 × 10^2^ M^−1^s^−1^ and the kd of 1.62 × 10^−2^ s^−1^, respectively (Figure 2B). Exifone did not exhibit a significant TDI effect, suggesting that it non-covalently binds to SARS-CoV-2 3CL^pro^ (Figure 2C). The compound showed non-competitive inhibition with an allosteric mechanism, with a *K*_i_ value of 1.58 μM (Figure 2D). However, the IC_50_ value of benserazide hydrochloride showed a remarkable twenty-fold decrease from 7.55 μM at 3 min to 0.37 μM at 30 min, suggesting its covalent interaction with SARS-CoV-2 3CL^pro^ (Figure 2E). Benserazide hydrochloride exhibited time and concentration-dependent inhibition, with *K*_I_ and *k*_inact_ values being 2.24 μM and 0.08 s^−1^, respectively (Figure 2F,G). Regarding safety profiles, we assessed the cytotoxicity of both compounds on Vero-E6 cells, which are typically employed for viral infection studies. The cytotoxicity assay revealed that exifone and benserazide hydrochloride, with a half-maximal cytotoxic concentration (CC_50_) exceeding 100 μM, were generally well tolerated, suggesting a favorable safety profile (Figure 2H,I).

### 2.3. Binding Modes of Exifone and Benserazide Hydrochloride with SARS-CoV-2 3CL^pro^

**Figure 3:**
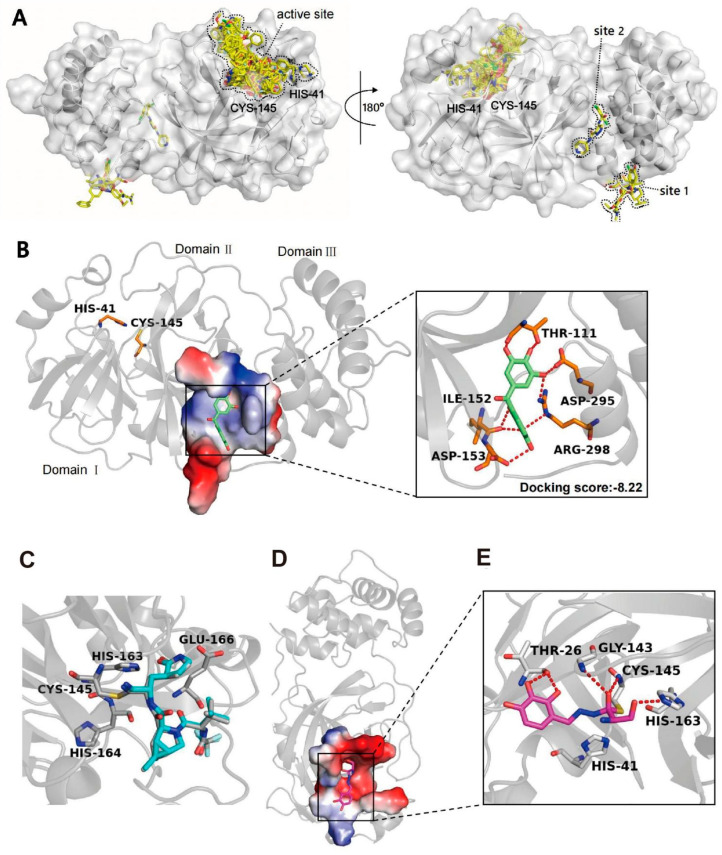
Binding modes of exifone and benserazide hydrochloride to SARS-CoV-2 3CL^pro^. (**A**) Distribution of inhibitor binding sites on 3CL^pro^. Catalytic residues His-41 and Cys-145, the active site, and two allosteric drug binding sites are highlighted. The inhibitors are depicted as yellow sticks. (**B**) Predicted binding mode of exifone with SARS-CoV-2 3CL^pro^ (PDBID:7AGA). Exifone is depicted as green sticks, and key residues are colored in orange. Hydrogen bonds are shown as red dashes. (**C**) The re-docking was performed using the structure model of SARS-CoV-2 3CL^pro^ (PDBID: 7SI9). The native ligand is depicted as cyan sticks. (**D**) Overview of the conformation of benserazide hydrochloride combined with SARS-CoV-2 3CL^pro^. (**E**) 3D receptor-ligand interactions analysis of benserazide hydrochloride combined with SARS-CoV-2 3CL^pro^. The protein secondary structure is shown in gray. Hydrogen bonds are shown in red dashes, and key residues are labeled.

To figure out the binding modes of exifone and benserazide hydrochloride with SARS-CoV-2 3CL^pro^, we preferentially selected all available crystal structures of SARS-CoV-2 3CL^pro^ with its inhibitors from PDB, which were superimposed in order to choose an appropriate protein model. As demonstrated in Figure 3A, the majority of inhibitors were found to bind at the active site of the enzyme, while others bound to allosteric sites distinct from the active site, specifically sites 1 and 2. Subsequently, molecular docking simulations were performed at these two sites. The result showed that exifone binds at the cleft between domain II and domain III within SARS-CoV-2 3CL^pro^, with a docking score of −8.22 kcal/mol. As shown in Figure 3B, exifone mainly interacts with SARS-CoV-2 3CL^pro^ through hydrogen bonds, forming eight hydrogen bonds with five residues, including T111, I152, D153, D295, and R298. R298 has been reported to be crucial to dimerization [45]. The mutation R298A caused a reorientation of the dimerization domain relative to the catalytic domain, leading to the destabilization of the active pocket [46]. El-Baba et al. suggested that a highly reactive pocket in the dimerization region at the domain III apex of SARS-CoV-2 3CL^pro^ has been recognized as a possible allosteric site [46]. Interestingly, our docking results revealed that exifone is located near this pocket and forms hydrogen bonds with key residues D295 and R298.

Similarly, molecular docking was conducted to further explore the inhibitory mechanism of benserazide hydrochloride against SARS-CoV-2 3CL^pro^. Before docking benserazide hydrochloride with 3CL^pro^, we first conducted re-docking on the crystal structure of 3CL^pro^ (PDB: 7SI9) to validate our docking method. The docking pose of the cognate ligand was nearly identical to that observed in the crystal structure (Figure 3C, RMSD = 0.62 Å), indicating that our docking simulation accurately reproduces the binding mode of the native ligand. As shown in Figure 3D, benserazide hydrochloride is well accommodated in the catalytic pocket of SARS-CoV-2 3CL^pro^. Two phenolic hydroxyl groups of benserazide hydrochloride form two hydrogen bonds with residue T26. Additionally, SARS-CoV-2 3CL^pro^ employs a non-canonical catalytic dyad, which consists of residues C145 and H41, along with the main chain amide NH groups of G143, S144, and C145, to create oxygen anion holes [47]. Remarkably, benserazide hydrochloride forms two hydrogen bonds with the oxyanion hole of the catalytic site to prevent effective cleavage of the substrate by 3CL^pro^ (Figure 3E). In addition, its terminal hydroxyl group forms a hydrogen bond with H163. The molecular docking suggested a covalent binding consistent with the experimental results between benserazide hydrochloride and C145. Therefore, the way that benserazide hydrochloride occupies the binding pocket can lead to inhibition of the activity against SARS-CoV-2 3CL^pro^.

### 2.4. Exifone and Benserazide Hydrochloride Exhibiting Broad-Spectrum Inhibition Against 3CL^pro^ of Various Coronaviruses

**Figure 4:**
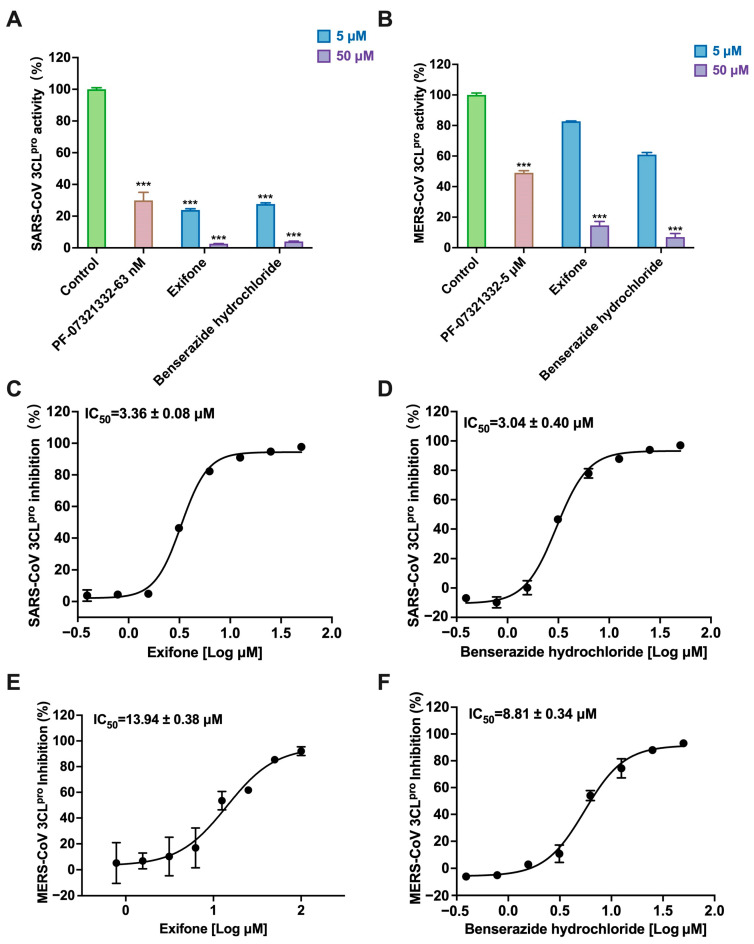
Selectivity of exifone and benserazide hydrochloride on viral proteases. (**A**) This panel shows the extent to which exifone and benserazide hydrochloride inhibit SARS-CoV 3CL^pro^. (**B**) The figure depicts the inhibitory action of exifone and benserazide hydrochloride on MERS-CoV 3CL^pro^. (**C**,**D**) The IC_50_ values for the inhibition of SARS-CoV 3CL^pro^ by exifone and benserazide hydrochloride are presented. (**E**,**F**) The IC_50_ values are shown for the inhibition of MERS-CoV 3CL^pro^ by compounds. Each data point represents the mean ± SEM of three independent experiments. Statistical differences were analyzed and determined based on *p*-values (*** *p* < 0.001).

Considering that the 3CL proteases of various coronaviruses share some sequence homology, we also determined the inhibitory activities of exifone and benserazide hydrochloride against 3CL^pro^ of other coronaviruses, specifically SARS-CoV and MERS-CoV. Encouragingly, both compounds demonstrated inhibitory effects on the 3CL^pro^ of these viruses as illustrated in Figure 4A,B. For comparison, PF-07321332, a known broad-spectrum 3CL^pro^ inhibitor, was used as a positive control. Further analysis revealed that exifone maintained a similar IC_50_ value of 3.36 ± 0.08 μM against SARS-CoV 3CL^pro^ as observed with SARS-CoV-2 3CL^pro^ (Figure 4C). However, compared with exifone, benserazide hydrochloride exhibited a decreased inhibitory effect (IC_50_ = 3.04 ± 0.40 μM) (Figure 4D). Although the inhibitory effects of exifone and benserazide hydrochloride on MERS-CoV 3CL^pro^ were diminished to 13.94 ± 0.38 μM and 8.81 ± 0.34 μM, respectively (Figure 4E,F), the results support the conclusion that the two compounds possess broad-spectrum inhibitory properties against 3CL^pro^ of different coronaviruses.

### 2.5. Evaluation of Host Protease Selectivity of Exifone and Benserazide Hydrochloride

**Figure 5:**
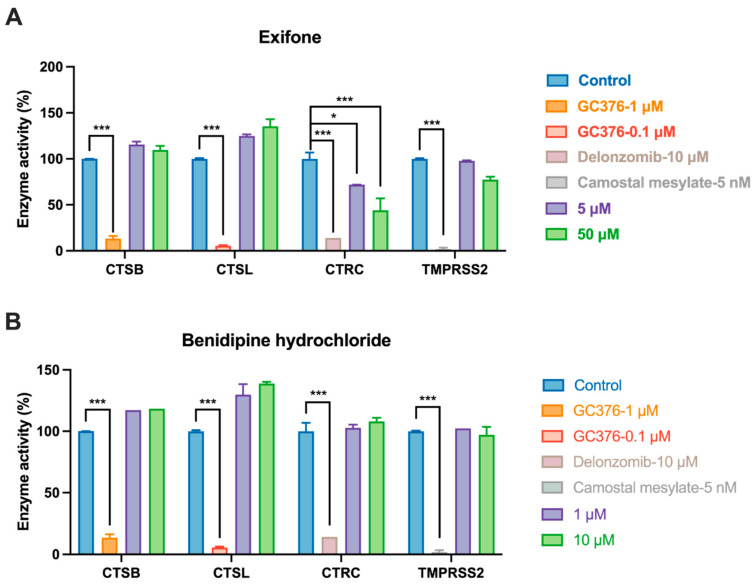
Selectivity of compounds in inhibiting host proteases. (**A**,**B**) The figure depicts the inhibitory activity of exifone and benserazide hydrochloride against CTSB, CTSL, and CTRC. Each data point represents the mean ± SEM of three independent experiments. Statistical differences were analyzed and determined based on *p*-values (* *p* < 0.05 and *** *p* < 0.001).

The specificity of a drug’s target is essential for clinical success, as off-target interactions often lead to therapeutic failure [48]. Therefore, we assessed the selectivity of our compounds against four host proteases: CTSB, CTSL, CTRC, and TMPRSS2. Our studies indicated that neither compound significantly inhibited CTSB, CTSL, and TMPRSS2 (Figure 5). GC-376, which inhibits CTSB and CTSL, was used as a positive control. Delonzomib, recognized for its inhibitory action on the chymotrypsin-like activity of the proteasome, served as an additional positive control of CTRC [49]. As shown in Figure 5A, exifone demonstrated significant inhibitory activity against CTRC concentrations of 5 and 50 μM (*p* < 0.05), whereas benserazide hydrochloride did not (Figure 5B). Given these findings, benserazide hydrochloride displays a promising safety profile, justifying further investigation as a potential 3CL^pro^ inhibitor.

### 2.6. Exifone’s Inhibitory Activity on Wild and Mutant SARS-CoV-2-S Pseudovirus

**Figure 6:**
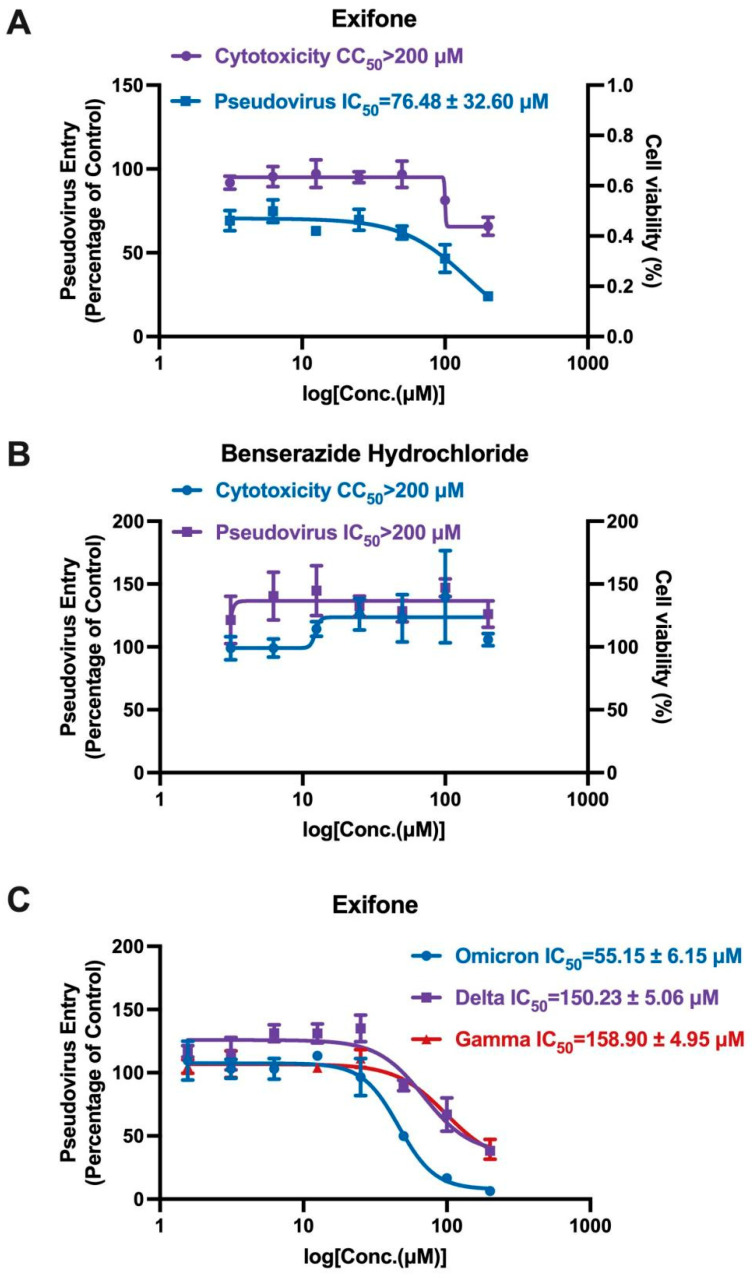
Evaluation of compound activity against SARS-CoV-2-S pseudovirus. (**A**,**B**) These pictures illustrate the antiviral activity and cytotoxicity profiles of exifone and benserazide hydrochloride, providing insights into their effectiveness and safety profiles. (**C**) The diagram specifically highlights exifone’s ability to inhibit the infection of hACE2-HEK293T cells by diverse strains of SARS-CoV-2-S pseudovirus. Each data point represents the mean ± SEM of three independent experiments.

In our quest to identify antiviral compounds capable of dual-targeting both the 3CL^pro^ and the ACE2/S-RBD interface, we utilized a lentiviral system to generate a series of wild and mutant pseudoviruses, including strains Delta, Gamma, and Omicron. As depicted in Figure 6A, exifone exhibited dose-dependent inhibition of the SARS-CoV-2-S pseudovirus, with an IC_50_ value of 76.48 ± 32.60 μM. The compound’s CC_50_ exceeded 200 μM, signifying low cytotoxicity against hACE2-HEK293T cells was low. Conversely, benserazide hydrochloride lacked the capacity to inhibit pseudovirus entry into host cells, as illustrated in Figure 6B. These findings indicated that exifone also exerted antiviral effects against the mutant pseudotyped SARS-CoV-2 strains infecting hACE2-HEK293T cells. Notably, the inhibitory effect of exifone on the Omicron strain paralleled that of the wild type, with an IC_50_ value of 55.15 ± 6.15 μM (Figure 6C). A comprehensive comparison of the antiviral activities of exifone and benserazide hydrochloride, as detailed in Table 3, revealed that exifone displayed robust inhibitory activity in both 3CL^pro^ enzyme inhibition and pseudovirus infection assays. In contrast, benserazide hydrochloride exhibited relatively weaker inhibitory effects across these assays.

### 2.7. Mechanistic Insights into Exifone’s Inhibitory Effect on Pseudovirus Entry into Cells

**Figure 7:**
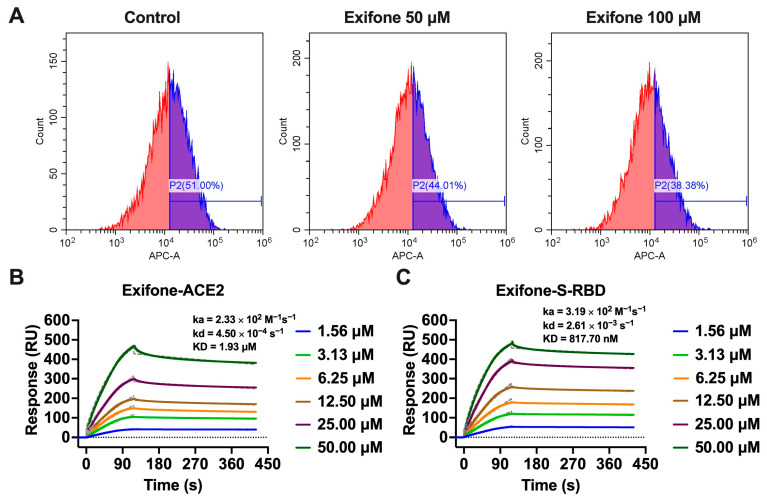
Mechanisms of exifone blocking the ACE2/S-RBD interaction. (**A**) The binding of soluble his-S-RBD to Vero-E6 cells was measured by flow cytometry with specific mean fluorescence intensities indicated within the panels. (**B**,**C**) The subsequent panels present the SPR binding curves, delineating the interactions of exifone with both the wild-type S-RBD and ACE2.

The S-RBD is instrumental in facilitating viral entry into host cells by engaging with ACE2 on the host’s plasma membrane. To elucidate the mechanism by which exifone impedes pseudovirus entry, a flow cytometry blocking assay was employed. This assay aimed to ascertain whether exifone could disrupt the binding of his-S-RBD to Vero-E6 cells, which natively express the ACE2 receptor. The addition of exifone at concentrations of 50 and 100 µM to the soluble S-RBD binding assay led to a marked reduction in the S-RBD binding signal (Figure 7A), underscoring the potent inhibitory effect of exifone on S-RBD binding to Vero-E6 cells. Additionally, the binding affinity of exifone to S-RBD or ACE2 was evaluated using the SPR assay. Exifone exhibited dose-dependent binding to the immobilized ACE2 protein with a KD value of 1.93 μM, ka and kd values of 2.33 × 10^2^ M^−1^s^−1^ and 4.50 × 10^−4^ s^−1^, respectively. Exifone displayed slow binding kinetics (ka = 3.19 × 10^2^ M^−1^s^−1^) and slow dissociation (kd = 2.61 × 10^−3^ s^−1^) to both S-RBD and ACE2. Moreover, exifone exhibited faster kinetics and higher affinity for SARS-CoV-2 S-RBD compared to human ACE2 (Figure 7B,C).

### 2.8. Binding Modes of Exifone with S-RBD of SARS-CoV-2 Wild-Type and Three Variants

**Figure 8:**
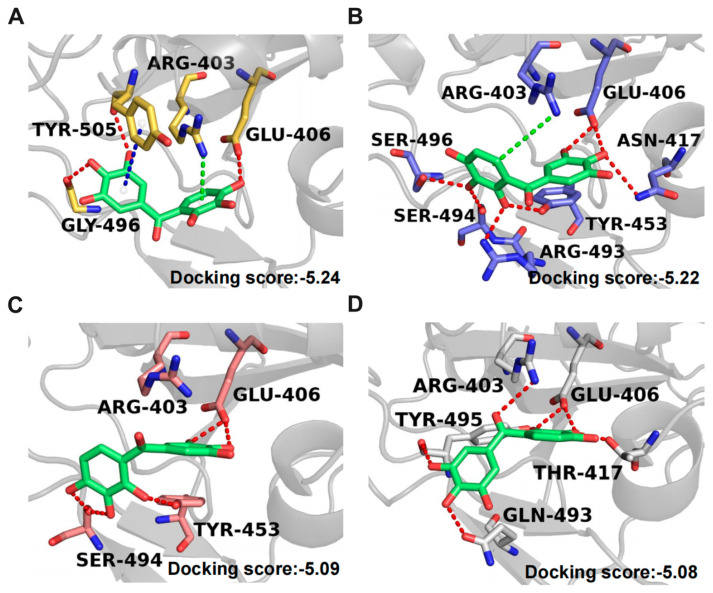
Predicted binding modes of exifone with wild type and three mutant SARS-CoV-2 S-RBDs. Key residues on the SARS-CoV-2 wild-type (**A**), Omicron S-RBD (**B**), Delta S-RBD (**C**), and Gamma S-RBD (**D**) are depicted as sticks and labeled appropriately. Red, blue, and green dashes represent hydrogen bonds, pi-pi stacking, and pi-cation interactions, respectively. The corresponding average docking scores are provided in the bottom right corner of each graph.

Because exifone can inhibit SARS-CoV-2 by targeting multiple mutant S-RBDs, we conducted docking simulations on four additional proteins: the wild type and the Omicron, Delta, and Gamma variants of SARS-CoV-2. The best docking scores of exifone with these proteins are presented in Figure 8. In the wild-type S-RBD, the phenolic hydroxyl groups of exifone form hydrogen bonds with E406, G496, and Y505, respectively. Concurrently, one benzene ring of exifone engages in pi-pi stacking with Y505, and another forms a pi-cation interaction with R403. These residues are critical for interactions with ACE2 [50,51]. Moreover, exifone exhibits hydrophobic interactions with Y453 and Y495. In the Omicron S-RBD, exifone forms the largest number of hydrogen bonds compared to the other three variants. Additionally, one of its benzene rings interacts with R403 through the pi-cation (Figure 8B). However, in the Delta variant, we observed no interaction between exifone and R403 (Figure 8C). In the Gamma variant, the pyrogallol group of exifone is positioned away from R403 (Figure 8D), in the opposite orientation to that in the wild-type and Omicron variant. This positioning may not prevent R403 from swinging and potentially contacting ACE2. The distinct binding modes observed could account for exifone’s reduced efficacy in inhibiting the Delta and Gamma variants.

## 3. Materials and Methods

### 3.1. Cell Culture

HEK293T cells overexpressing human ACE2 receptor (hACE2-HEK293T) have been constructed in our laboratory using the method described previously [52]. African green monkey kidney epithelial cells Vero-E6 and HEK293F cells were obtained from the Type Culture Resource Bank of the Chinese Academy of Sciences (Shanghai, China). All adherent cells were routinely placed in DMEM medium supplemented with 10% fetal bovine serum (FBS) (Yeasen Biotechnology, Shanghai, China), 100 U/mL penicillin, and 100 μg/mL streptomycin and stored at 37 °C in a humidified environment containing 5% CO_2_. HEK293F cells were grown in OPM-293 CD05 medium (OPM Biosciences, Shanghai, China) and incubated at 37 °C, 8% CO_2_, and 125 rpm with shaking.

### 3.2. Protein Expression and Purification

The expression and purification of SARS-CoV-2 3CL^pro^ followed the method previously established in our work [53]. In brief, the SARS-CoV-2 3CL^pro^ plasmid was transformed into *Escherichia coli* BL21 (DE3) as described earlier [54]. Protein expression was induced with 0.3 mM IPTG, followed by shaking incubation at 18 °C and 180 rpm for 16 h. The cells were harvested by centrifugation at 4000 rpm for 60 min at 4 °C. The pellet was lysed by the lysis composed of 25 mM Tris, 150 mM NaCl, 20 mM imidazole, 1 mM PMSF, and 1 mM DTT, and the lysate was subjected to centrifugation at 15,000 rpm for 30 min. The resulting supernatant was loaded onto Ni-NTA agarose (GE Healthcare, Chicago, IL, USA) and eluted with 300 mM imidazole. Further purification was achieved using a Superdex 200 10/300 column (GE Healthcare, USA). The purified protein was concentrated with a 10 kDa molecular weight cut-off concentrator and stored in a preservation buffer (25 mM HEPES, 150 mM NaCl, 1 mM DTT, pH 7.4) at −80 °C for subsequent experiments.

The cDNA fragment of S-RBD (residues 319 to 541) was cloned into a mammalian expression vector pTT5 with a 6 × His tag at the C-terminus. High-quality plasmids were transfected into HEK293F cells using PEI (Polysciences, Warrington, PA, USA). After 5 days of culture, the supernatant was collected, and the protein, His-tagged S-RBD (His-S-RBD), was purified using Ni-NTA (Smart-Lifesciences, Changzhou, China). The purity and accuracy of the purified His-S-RBD protein were confirmed by SDS-PAGE analysis.

### 3.3. Enzymatic Inhibition Assay

The 3CL^pro^ of SARS-CoV-2 or SARS-CoV was prepared as per the methods detailed in our previous studies [53,55,56]. The sources of the tested compounds were as follows: Vitamin K3 (VK3), delphinidin 3-sambubioside chloride, and norepinephrine were purchased from MedChemExpress (Shanghai, China); Exifone and dopamine hydrochloride were from Shanghai Yune Bio-Technology (Shanghai, China); Benserazide hydrochloride was purchased from Sparkjade Biotechnology Co., Ltd. (Shandong, China). In this assay, recombinant 3CL^pro^ at a final concentration of 120 nM was first mixed with the test compounds and incubated for 30 min. Subsequently, a fluorogenic substrate (Dabcyl-KNSTLQSGLRKE-Edans) supplied by Genscript (Nanjing, China) was added at a final concentration of 20 µM to initiate the enzymatic reaction. After incubation for 20 min, the fluorescence intensity was recorded at excitation and emission wavelengths of 340 nm and 490 nm, respectively, using a Cytation 5 plate reader (BioTek, Winooski, VT, USA). To determine the IC_50_ values, the assay was conducted at eight different concentrations of the test compounds, with each experiment replicated thrice. All the resulting data were analyzed using GraphPad Prism version 8.0 (San Diego, CA, USA).

A time-dependent inhibition (TDI) assay was performed consistent with the method reported in our previous publication [46]. SARS-CoV-2 3CL^pro^ was incubated with different concentrations of compounds in a suitable reaction buffer for two distinct durations: 3 min and 30 min, respectively.

In the MERS-CoV 3CL^pro^ inhibition assays, the protease at a final concentration of 2 µM was incubated with various test compounds for a duration of 30 min. Following this incubation period, the fluorogenic substrate was added at a concentration of 40 µM to trigger the reaction, which was then allowed to proceed for 60 min. The fluorescence signal was recorded under the same conditions as those used for the SARS-CoV-2 3CL^pro^ assay. The data from all experiments were systematically processed and analyzed using GraphPad Prism 8.0 (San Diego, CA, USA).

### 3.4. SPR

The binding affinity of compounds to SARS-CoV-2 3CL^pro^ was evaluated through surface plasmon resonance (SPR) using a Biacore T200 instrument (Cytiva, Marlborough, MA, USA). SARS-CoV-2 3CL^pro^ was immobilized onto a CM5 chip by amine coupling in sodium acetate buffer (pH 5.5), with a flow rate of 10 μL/min. Following chip activation with a mixture of NHS and EDC, 30 μg/mL of SARS-CoV-2 3CL^pro^ was injected, and the surface was subsequently blocked with ethanolamine. Solutions of compounds, prepared with 5% DMSO, were injected at varying concentrations, and the interaction was monitored during 60 s association and dissociation phases. The experiments were conducted in PBS buffer (0.05% Tween-20, 5% DMSO, pH 7.4) at 25 °C. To account for changes in refractive index and data drift, appropriate corrections were applied. Binding affinities were determined by fitting the data to a Langmuir 1:1 binding model using Biacore Evaluation software.

Similarly, the binding affinity of exifone and benserazide hydrochloride to ACE2 and S-RBD was assessed using SPR on the same Biacore T200 instrument (Cytiva, USA). ACE2 and S-RBD were separately immobilized onto a CM5 chip via amine coupling in sodium acetate buffer (pH 4.0 for ACE2 and pH 5.5 for S-RBD) at a flow rate of 10 μL/min. After the chip surface was activated with NHS and EDC, ACE2 and S-RBD were injected at concentrations of 20 μg/mL and 30 μg/mL, respectively, and the surface was blocked using ethanolamine. Exifone and benserazide hydrochloride solutions, containing 5% DMSO, were injected at different concentrations, and the binding interactions were evaluated during 120 s association and 300 s dissociation phases. All measurements were performed in PBS buffer (0.05% Tween-20, 5% DMSO, pH 7.4) at 25 °C, with corrections applied to address refractive index shifts and data drift. The binding affinities were quantified by fitting the interaction data to a Langmuir 1:1 binding model using Biacore Evaluation software.

### 3.5. Determination of k_inact_/K_I_ Values

The *k*_inact_/*K*_I_ values were calculated based on the methodology described in our previous publication [19]. SARS-CoV-2 3CL^pro^ (120 nM) was incubated with different concentrations of compounds at 37 °C for time intervals of 0, 10, 20, and 30 min. After incubation, a substrate solution (20 μM) was added to start the enzymatic reaction, and fluorescence was monitored using a Cytation 5 plate reader (BioTek, Winooski, VT, USA), with an excitation wavelength of 340 nm and emission at 490 nm. Enzyme activity was then measured to assess the relationship between inhibitor concentration and the reaction rate. The data were fitted to Michaelis–Menten kinetics to determine the inhibitor constant (*K*_I_). Furthermore, enzyme activity was measured at various time points using a fixed inhibitor concentration. The data were analyzed by fitting the time-dependent activity to a first-order kinetic model to derive the inactivation rate constant *(k*_inact_). Finally, the *k*_inact_/*K*_I_ value was calculated.

### 3.6. Cell Viability Assay

Vero-E6 cells were seeded in 96-well plates and cultured overnight. Afterwards, different concentrations of the test compounds were added to the cells. After 24 h of incubation with the compounds, a 10% Cell Counting Kit-8 reagent (CCK-8, Meilunbio, Dalian, China) was introduced to each well, and the cells were further incubated for 1 h. The viability of the cells was then assessed by measuring the optical density at 450 nm using a Cytation 5 plate reader (BioTek, Winooski, VT, USA).

In the cytotoxicity assay for hACE2-HEK293T cells, which aimed to rule out false-positive results in the pseudovirus neutralization assay, the drug treatment method and incubation time were kept consistent with those used in the pseudovirus neutralization assay. A 50 μL suspension of 5 × 10^5^ cells per well was added to each well of a 96-well plate and allowed to adhere overnight. The following day, the cells were pretreated with 10 μL of 10 × concentrated test compounds for 1 h at 37 °C, after which 40 μL of medium was added to each well. After a 24-h incubation period, the medium was replaced, and the cells were incubated for an additional 24 h. The medium was then replaced with CCK-8 solution, prepared according to the manufacturer’s instructions. The cytotoxic effects of the compounds were quantified by measuring absorbance at 450 nm using a Cytation 5 plate reader (BioTek, Winooski, VT, USA).

### 3.7. Molecular Docking

#### 3.7.1. Searching for Allosteric Sites of SARS-CoV-2 3CL^pro^

All crystal structures of SARS-CoV-2 3CL^pro^ with co-crystallized binders were downloaded from the Protein Data Bank (PDB), which were provided as a table in the supplementary data. Subsequently, all of the crystal structures of the inhibitor-bound SARS-CoV-2 3CL^pro^ were collected for a comparative analysis. To identify the potential allosteric sites, 134 co-crystallized inhibitors of SARS-CoV-2 3CL^pro^ from PDB were superimposed using chain A of 6LZE as the reference structure, which were performed and visualized in PyMOL (The PyMOL Molecular Graphics System version 2.5, Schrödinger, LLC, NY, USA). The structure 6LZE was selected for superposition of the available 3CL^pro^ crystal structures in the PDB, as this structure has a high resolution of 1.5 Å and complete amino acid coordinates and has been widely used as a receptor template in previous studies [57,58]. Based on the alignment, the two protein structures that have the inhibitors bound at the sites outside the active site, namely, 7ABU and 7AGA, were chosen for the docking of exifone with the 3CL^pro^.

#### 3.7.2. Ligand Preparation

The 3D structures of exifone and benserazide hydrochloride were obtained from the NCBI PubChem compound database (https://pubchem.ncbi.nlm.nih.gov, accessed on 1 October 2024). Both molecules were prepared with LigPrep (Schrödinger, LLC) to generate all stereoisomers and different protonation states with Epik [59].

#### 3.7.3. Protein Model Preparation

The crystal structures of 7AGA, 7ABU, and 7SI9 were downloaded from PDB as the receptors of SARS-CoV-2 3CL^pro^. The first two proteins were chosen as the models for allosteric site docking, and the last one was used as the model for covalent docking. Each protein was prepared with Protein Preparation Wizard Workflow in Maestro (Maestro, Version 12.8; Schrodinger, LLC) at a pH of 7.0 ± 2.0. Other parameters were set as the default. The hydrogen bond network of the protein was optimized, and the protein structure was further energy-minimized by the OPLS4 force field.

The crystal structures of 6M0J, 7XAZ, 7V8B, and 7NXC were downloaded from PDB as the receptors of the SARS-CoV-2 wild-type and the three mutants. Among them, 6M0J is the S-RBD of wild-type SARS-CoV-2 in complex with ACE2, while 7XAZ, 7V8B, and 7NXC represent the S-RBD of Omicron, Delta, and Gamma variants, respectively. Each protein was prepared with the same procedure as the SARS-CoV-2 3CL^pro^ mentioned above.

#### 3.7.4. Docking of Exifone with SARS-CoV-2 3CL^pro^ and Four S-RBDs

The molecular docking was accomplished by Glide [60]. In SARS-CoV-2 3CL^pro^, the grid box was set to 25 Å × 25 Å × 25 Å centered at the co-crystallized ligand. The Glide XP [61] mode was used to dock exifone into two potential allosteric sites. The pose with the best docking score was used for further analysis.

It has been reported that K417 of S-RBD forms a salt bridge and a hydrogen bond interaction with D30 of ACE2 [62]. Y505 forms two hydrogen bonds with E37 and R393 of ACE2, and it also interacts with the hydrophobic part of K353 of ACE2 [62]. Meanwhile, Q493 and Y449 are important residues that bind to ACE2 and form two hydrogen bonds with ACE2 [62]. Therefore, these four residues, Lys417, Tyr449, Gln493, and Tyr505, were taken as the grid center of 6M0J. The grid box was set to 35 Å × 35 Å × 35 Å. The SP [61] mode of Glide was used to dock exifone into the S-RBD binding site. The docking scoring function was used to rank all outputs. Finally, the pose with the best docking score was retained for further analysis.

#### 3.7.5. Docking of Benserazide Hydrochloride with SARS-CoV-2 3CL^pro^

Covalent docking was conducted with default options using the module “covalent docking” in Glide [63]. Cys145 was defined as a reactive residue. The centroid of the active site for the docking was defined as the center of mass of the ligand in 7SI9, and the box size was automatically set to be similar to the ligand. Subsequently, “Nucleophilic Addition of Double Bonds” was chosen as the reaction type, and “Pose Prediction (Thorough)” was chosen as the docking mode to achieve accurate pose prediction. All results were visualized with PyMOL.

### 3.8. Target Selectivity Tests Toward Host Proteases

Target selectivity tests toward host proteases were conducted based on our previous method [55,56]. Cathepsin B (CTSB) (SinoBiological, Beijing, China) at a final concentration of 100 nM was prepared in buffer with 20 mM sodium acetate, 1 mM EDTA, and 2 mM DTT (pH 5.5) and incubated for 30 min at 30 °C. Next, the working solution of CTSB was diluted into 500 pM with the reaction buffer (100 mM MES pH 6.0, 1 mM EDTA, 2 mM DTT, and 0.01% Tween-20) and kept for 30 min. Then, the FRET substrate Z-Phe-Arg-AMC (NJPeptide, Nanjing, China) at 5 μM was added to activate the enzymatic reaction. The fluorescence signals were measured using a Cytation 5 plate reader (BioTek, Winooski, VT, USA) at 360 nm (excitation)/460 nm (emission) for 20 min.

Cathepsin L (CTSL) (SinoBiological, Beijing, China) at a final concentration of 100 nM in buffer (20 mM sodium acetate, 1 mM EDTA, and 5 mM DTT, pH 5.5) was activated by incubation at 30 °C. Then, the working solution of CTSL was diluted into 300 pM with the reaction buffer and incubated for 30 min. The addition of 5 μM FRET substrate Z-Phe-Arg-AMC (NJPeptide, Nanjing, China) initiated the reaction. The fluorescence signals at 360 nm (excitation)/460 nm (emission) were measured for 20 min with the Cytation 5 plate reader (BioTek, Winooski, VT, USA).

Chymotrypsin (CTRC) (Novoprotein, Suzhou, China) at a final concentration of 5 μg/mL was incubated in a buffer solution containing 50 mM Tris, 10 mM CaCl_2_, and 150 mM NaCl at pH 7.5 for a period of 30 min. Subsequently, trypsin was added to the mixture at the same concentration (5 μg/mL) and incubated for an additional 60 min at 37 °C. Following this, the reaction was initiated by adding 100 µM of the Succinyl-Ala-Ala-Pro-Phe-p-nitroanilide substrate (NJPeptide, Nanjing, China), and the mixture was incubated for another 60 min at 37 °C. Finally, the activity of CTRC was quantified by measuring the absorbance at 405 nm using a Cytation 5 plate reader (BioTek, Winooski, VT, USA).

TMPRSS2 protease at the concentration of 5 nM (MedChemExpress, Shanghai, China) was incubated with compounds in the reaction buffer (50 mM Tris-HCl, 1 mM CaCl_2_, 0.1% Tween 20, pH 8.0) for 1 h at 37 °C. Then, 45 μM substrate Boc-GIn-Ala-Arg-AMC (NJPeptide, Nanjing, China) was added to initiate the reaction. Fluorescence intensity was detected using a Cytation 5 plate reader at 380 nm (excitation)/460 nm (emission) for 1.5 h.

### 3.9. Pseudovirus Production

The method was described in our previous article [50,52]. HEK293T cells were cultured in 100 mm cell culture dishes, and the confluence degree was about 50–70% before transfection. The medium was replaced with DMEM basic medium. Plasmid-transfection reagent complex was prepared by adding 1 mL medium and 30 μg transfection reagent Lipofiter 3.0 (Hanbio, Shanghai, China) to centrifuge tube A and incubating for 5 min. In centrifuge tube B, 1 mL of culture medium, 12 μg of pNL4-3.luc.R.E plasmid (gifted from Prof. Lu Lu, Fudan University), and 3 μg of pcDNA3.1-SARS-CoV-2-Spike plasmid (Precedo, Anhui, China) or other plasmids of mutant strains were added. The optimized expression plasmid encoding the full-length Omicron SARS-CoV-2 S protein was obtained from Genscript (Nanjing, China). Plasmids expressing Delta (Cat. No. #172320) and Gamma (Cat. No. #172320) S proteins were from Addgene (Watertown, MA, USA). Then, the liquid in centrifuge tube A was transferred to centrifuge tube B and then mixed, incubated for 20 min, and slowly added to the cell culture medium by dripping and gently shaking. After 6–8 h of transfection, the medium was replaced with DMEM medium containing serum. The viral supernatant was collected at 48 h after transfection, filtered through a 0.45 μm filter membrane, and aliquots of 1 mL per tube were frozen at −80 °C.

### 3.10. Pseudovirus Neutralization Assay

A pseudovirus neutralization assay was performed according to our previously reported method [50,52]. The hACE2-HEK293T cells were seeded at a density of 50 μL/well and 2.5 × 10^4^ cells/well in white background 96-well plates (Biosharp, Hefei, China) and cultured overnight. Ten μL of 10 × corresponding concentration of compounds were added to each well and incubated for 1 h at 37 °C. The pseudovirus was gently blown and mixed, and 40 μL was added to each well. In the negative control group, 40 μL of culture medium was added. After 24 h of infection, the medium was replaced. After 48 h, the supernatant was discarded. Then, 20 μL of 1× lysis buffer prepared by diluting the 5 × lysis solution from the firefly luciferase reporter gene assay kit (Meilunbio, Dalian, China) was added to each well. The cells were lysed on a shaker for 30 min. Next, 100 μL of luciferase detection reagent was added to each well, and luminescence was measured immediately using a Multilabel Reader (SpectraMax Paradigm, San Jose, CA, USA). Relative luminescence (%) was calculated according to the manufacturer’s instructions. The IC_50_ values were calculated using non-linear regression analysis with GraphPad Prism 8.0 (CA, USA).

### 3.11. Flow Cytometry Detecting the Block Activity of the Test Compounds Against SARS-CoV-2 S-RBD/ACE2 Interaction

Vero-E6 cells were counted after digestion from the dish and were aliquoted into tubes at a concentration of 3 × 10^6^ cells/mL, with 50 μL per tube. After preincubating 25 μL of his-S-RBD at a concentration of 2 μM with 25 μL of each candidate solution for 1 h, the Vero-E6 cells were then incubated with the mixture for 1 h at room temperature. Following this, the cells were washed twice with PBS. Subsequently, 100 μL of CoraLite^®^ Plus 647-conjugated 6 × His, His-tag Monoclonal antibody (Proteintech, Wuhan, China) was added at a 1:400 dilution. The cells were then incubated with the antibody at 4 °C for 30 min and washed twice before proceeding to flow cytometry analysis (Beckman, San Jose, CA, USA).

## 4. Conclusions and Discussion

Emerging coronaviruses, such as SARS-CoV, MERS-CoV, and SARS-CoV-2, have caused global epidemics of severe human disease. These events have heightened awareness of the escalating risk of highly pathogenic coronavirus infections and have underscored the urgent need for the development of effective anti-coronavirus drugs. The 3CL^pro^ enzyme is a highly conserved cysteine protease that is vital to the coronavirus life cycle and represents one of the most promising targets for antiviral drug development [64]. Meanwhile, the ACE2/S-RBD binding site is a critical step in the viral infection of human cells [65]. Thus, for COVID-19, therapeutic regimens that simultaneously target both 3CL^pro^ and the ACE2/S-RBD interface may offer greater advantages. These dual-target approaches can reduce the polypharmacy burden on the host system by minimizing the necessity for multiple combination drugs.

This study underscores the role of bioactive polyphenols, particularly exifone and benserazide hydrochloride, in inhibiting the activity of SARS-CoV-2 3CL^pro^ (Figure 1). The mechanisms of action of these compounds are thoroughly detailed. In this study, exifone does not exhibit significant TDI effects, whereas benserazide hydrochloride demonstrates pronounced TDI characteristics, as shown in Figure 2. Additionally, the binding modes of these compounds with SARS-CoV-2 3CL^pro^ are elucidated, with exifone binding at a cleft between domain II and III within 3CL^pro^, primarily through hydrogen bonds, while benserazide hydrochloride is accommodated in the catalytic pocket of the enzyme (Figure 3). Exifone and benserazide hydrochloride showed the broad-spectrum inhibitory effects on various coronaviral 3CL^pros^, including those of SARS-CoV and MERS-CoV (Figure 4). The specificity of these compounds for their targets, their impact on host proteases, and their safety profiles were explored, with particular emphasis on benserazide hydrochloride’s favorable safety profile as a potential 3CL^pro^ inhibitor (Figure 5). Benserazide hydrochronide is an inhibitor of aromatic L-amino acid decarboxylase (also known as dopa decarboxylase), and it is used in conjunction with for the treatment of Parkinson’s disease [66]. Currently, studies suggest that benserazide hydrochronide is a potential therapeutic agent for the mutant H7N9 virus, as indicated by molecular docking and molecular dynamics simulations [67,68].

In addition to its inhibitory effects on 3CL^pro^, exifone demonstrated antiviral activity against various SARS-CoV-2 pseudovirus strains. It inhibited pseudovirus entry into hACE2-HEK293T cells in a dose-dependent manner, with an IC_50_ of 76.48 ± 32.60 µM for the wild-type strain and 55.15 ± 6.15 µM for the Omicron strain, indicating its effectiveness against both (Figure 6A,C). Exifone exhibited low cytotoxicity, with a CC_50_ exceeding 200 µM (Figure 6B). Mechanistic studies revealed that exifone blocks the interaction between S-RBD and ACE2, as confirmed by flow cytometry and SPR assays (Figure 7). Docking simulations further elucidated distinct binding modes of exifone with the S-RBD of different variants, providing insights into its reduced efficacy against Delta and Gamma strains (Figure 8).

The dual-target therapeutic strategy that blocks both 3CL^pro^ and the ACE2/S-RBD interaction is suggested to be more advantageous in reducing the polypharmacy burden on the host system. Despite some limitations, the potential of exifone in clinical use is also highlighted. Exifone, a member of the benzophenones, was first discovered to aid in the treatment of microcirculation disorders [69]. Subsequently, it was shown to exhibit neuroprotective activity across a range of preclinical models [70]. Through controlled clinical trials, oral doses ranging from 200 to 600 mg/day were demonstrated to be effective for treating elderly patients with Alzheimer-type dementia and cognitive deficits associated with Parkinson’s disease, and it was approved as a drug in 1988 [71]. Despite its antiviral potential, exifone has been withdrawn from the market due to hepatotoxicity [44]. However, its antiviral activity has reignited interest in exploring its therapeutic potential. Future research should focus on elucidating the molecular mechanisms underlying its hepatotoxicity and identifying strategies to mitigate this adverse effect while preserving its antiviral activity.

More importantly, the presence of the polyhydroxyphenol moiety appears to confer some advantages in inhibiting 3CL^pro^, particularly when considered alongside the results presented here and elsewhere [25,27]. Specifically, the triphenol compounds have demonstrated stronger inhibitory activity compared to those containing only two phenolic hydroxyl groups. These findings may offer valuable insights for the rapid drug discovery process in the event of future coronavirus pandemics. Future efforts should aim to optimize the structure of these compounds to improve their efficacy and reduce toxicity.

In summary, this study provides valuable insights into the antiviral potential of bioactive polyphenols and highlights the need for further research to optimize their therapeutic applications.

## Figures and Tables

**Table 3:** CC_50_/IC_50_ values for antiviral activity and cytotoxicity of compounds.

	3CL^pro^ Inhibition	Pseudovirus Activity Inhibition	Cell Viability Assay	
Type of Experiments			Vero-E6	hACE2-HEK293T
Compounds	IC_50_ (μM)		CC_50_ (μM)	
Exifone	3.18 ± 1.46	76.48 ± 32.60	>100	>200
Benserazide hydrochloride	0.37 ± 0.02	>200	>100	>200

## Data Availability

The raw data supporting the conclusions of this article will be made available by the authors on request.

## Supplementary Materials

The following supporting information can be downloaded at: https://www.mdpi.com/article/10.3390/ijms26052243/s1.
